# Simultaneous production of DHA and squalene from *Aurantiochytrium* sp. grown on forest biomass hydrolysates

**DOI:** 10.1186/s13068-019-1593-6

**Published:** 2019-10-29

**Authors:** Alok Patel, Ulrika Rova, Paul Christakopoulos, Leonidas Matsakas

**Affiliations:** Biochemical Process Engineering, Division of Chemical Engineering, Department of Civil, Environmental, and Natural Resources Engineering, Luleå University of Technology, 971 87, Luleå, Sweden

**Keywords:** Thraustochytrids, *Aurantiochytrium* sp., DHA, Squalene, Wood biomass, Organosolv pretreatment, Heterotrophic growth, Lipid production

## Abstract

**Background:**

Recent evidence points to the nutritional importance of docosahexaenoic acid (DHA) in the human diet. Thraustochytrids are heterotrophic marine oleaginous microorganisms capable of synthesizing high amounts of DHA, as well as other nutraceutical compounds such as squalene, in their cellular compartment. Squalene is a natural triterpene and an important biosynthetic precursor to all human steroids. It has a wide range of applications in the cosmetic and pharmaceutical industries, with benefits that include boosting immunity and antioxidant activity. Apart from its nutritional quality, it can also be utilized for high-grade bio-jet fuel by catalytic conversion.

**Results:**

In the present study, the potential of thraustochytrid strain *Aurantiochytrium* sp. T66 to produce DHA and squalene was evaluated. When the strain was cultivated on organosolv-pretreated birch hydrolysate (30 g/L glucose) in flask, it resulted in 10.39 g/L of cell dry weight and 4.98 g/L of total lipids, of which 25.98% was DHA. In contrast, when the strain was grown in a bioreactor, cell dry weight, total lipid, and DHA increased to 11.24 g/L, 5.90 g/L, and 35.76%, respectively. The maximum squalene yield was 69.31 mg/g_CDW_ (0.72 g/L) when the strain was cultivated in flask, but it increased to 88.47 mg/g_CDW_ (1.0 g/L), when cultivation shifted to a bioreactor.

**Conclusions:**

This is the first report demonstrating the utilization of low cost non-edible lignocellulosic feedstock to cultivate the marine oleaginous microorganism *Aurantiochytrium* sp. for the production of nutraceutical vital compounds. Owing to the simultaneous generation of DHA and squalene, the strain is suitable for industrial-scale production of nutraceuticals.

## Background

Algae-based renewable energy and chemicals have gained increasing attention over the past few decades, mostly due to their potential to lower greenhouse gas emissions and reduce our dependency on fossil fuels [1]. However, algae-based industries are still incapable of sustainable and cheap biofuel production, as external energy is required for upstream and downstream processing [2]. It has been argued that cost-effective production of biofuels from microalgae is possible only if it is integrated with co-production of value-added chemicals for the petrochemical or pharmaceutical industries [3]. Some microalgal species allow the co-production of value-added chemicals in the current biorefinery concept. Microalgae can be used for a variety of applications, such as renewable energy (bioethanol, biodiesel, and biogas), nutraceuticals (omega-3 and omega-6 fatty acids such as eicosapentaenoic acid (EPA), docosapentaenoic acid (DPA), docosahexaenoic acid (DHA), and functional proteins), pharmaceutical pigments (β-carotene, squalene, and astaxanthin), for food and cosmetics, fertilizers, and bioplastics [4]. This multiproduct paradigm makes them suitable for sustainable biorefinery concepts [3] and, particularly, the generation of high-value fatty acids.

Most naturally occurring fatty acids are composed of a 4–28 carbon atom unbranched chain; they can be saturated, monounsaturated, or polyunsaturated depending on the nature of the hydrocarbon chain [5]. The human body has the capacity to synthesize a variety of saturated and unsaturated fatty acids, but cannot synthesize some polyunsaturated fatty acids (PUFAs), such as omega-3 and omega-6 fatty acids, due to the lack of certain elongases (Elovl2 or Elovl5) and delta-6-desaturases (FADS2) [6]. However, the human body can synthesize the parent omega-3 fatty acids (α-linolenic acid; ALA) and omega-6 fatty acids (linoleic acid; LA) [7]. Although a small amount of LA can be converted to dihomo-γ-linolenic acid (DGLA) and arachidonic acid (AA), and a minimal amount of ALA can be converted to EPA and DHA, this is not nearly enough to cover the daily intake of 0.30–0.45 g EPA and DHA required by a male adult [8]. Hence, food-based intake of these essential fatty acids is paramount. Nowadays, the main source of PUFAs is fish belonging to the Salmonidae, Scombridae, and Clupeidae families, as they contain high levels of DHA and EPA [8]. Unfortunately, the rising demand for these fatty acids cannot be met solely by fish oil, because it is becoming increasingly environmentally unsustainable [9]. Other potential sources of PUFAs are vegetable oils and oleaginous microorganisms. Plants can employ the fatty acid synthase complex (desaturase enzyme) in plastids to synthesize certain PUFAs, such as oleic acid (18:1n9), LA (18:2n6), γ-LA (18:3n6), ALA (18:3n3), and octadecatetraenoic acid (18:4n3) [10]. However, plants cannot synthesize long-chain PUFAs as they lack separate desaturase/elongase enzymes [11]. Accordingly, elevated levels of long-chain omega-3 fatty acids, such as DPA, DHA, and EPA, can only be achieved with genetically engineered plants, such as *Brassica juncea*, *Brassica napus*, *Glycine max*, and *Arabidopsis thaliana*, following the introduction of desaturase/elongase genes [11, 12]. On the downside, plant cultivation is dependent on climate conditions and the availability of arable land. Importantly, the productivity of omega-3 fatty acids by plants is inferior to that achieved by oleaginous microorganisms [13]. Oleaginous microorganisms are natural producers of a diverse group of fatty acids, which vary depending on the chosen species and cultivation conditions [14]. Application of biochemical and genetic engineering techniques can further improve production of the desired compounds [15], which is important for the commercial production of microbial omega-3 fatty acids. The first known microbial strain for commercial γ-LA-rich oil production was the filamentous fungus *Mucor circinelloides* [16]. Several other microalgal species have been explored thereafter for commercial omega-3 PUFA production.

Microalgae such as diatoms are most commonly used for microbial production of DHA and EPA. The majority of diatoms can only grow photoautotrophically, which often hinders their commercialization due to low productivity. Heterotrophic growth, whereby microalgae grow on an organic carbon source such as glucose, can potentially increase PUFA productivity. To enable this, appropriate candidates capable of growth on organic carbon sources should be found. Thraustochytrids, a group of marine protists belonging to the Labyrinthula class of the Chromista Kingdom, can grow heterotrophically and can synthesize high amounts of DHA. These properties make them excellent candidates for commercial DHA production, with the *Aurantiochytrium*/*Schizochytrium* genera being of particular interest [17].

Most Thraustochytrids also possess significant quantities of natural antioxidants such as terpenoids, in their cellular compartment to protect omega-3 fatty acids from oxidative stress [18]. The most common terpenoid in *Aurantiochytrium*/*Schizochytrium* is squalene (2,6,10,15,19,23-hexamethyl-6,6,10,14,18,20-tetracosahexane), a polyunsaturated hydrocarbon triterpenoid [19]. Squalene is obtained mainly from the liver oil of deep-sea sharks and whales and acts as a precursor for the biosynthesis of bile acid, cholesterol, and steroids in animals and plants [20]. However, consumption of liver oil can pose a threat to our health, due to contamination with environmental pollutants, including heavy metals, mercury, and polychlorinated biphenyls along with a putrid odor and unpleasant taste [21, 22]. Moreover, the presence of chemically similar compounds such as cholesterol in liver oils can affect squalene purification. Squalene is used as a valuable ingredient in the cosmetic industry due to quenching of singlet oxygen (1O_2_) [23]. It is also used in the pharmaceutical industry, due to its positive effect on the immune response, the ability to reduce serum cholesterol levels and suppress tumor proliferation [24, 25], as well as an adjuvant to increase immune responsiveness to vaccines [26]. Squalene acts as a precursor of thousands of bioactive compounds, including sterols and hopanoids [27]. Another field of application of squalene is the production of high-grade bio-jet fuels by catalytic conversion. For example, in a study, the branched hydrocarbon of squalene was successfully converted into smaller hydrocarbon without skeletal isomerization and aromatization over ruthenium on ceria (Ru/CeO_2_) [28]. Similarly, pure squalene and squalene containing *Botryococcus braunii* liquid were utilized for bio-jet fuel production by Ru/CeO_2_ [29]. The global demand for squalene has been increasing over the past decade, amounting to around 2.67 kilotons in 2014 [30], and cannot be met solely by extracting it from the liver of marine animals; an approach that is severely affecting marine ecosystems. Only a handful of plant species are known to be capable of producing adequate quantities of squalene for pharmaceutical or nutraceutical industrial applications [31]. Instead, high-scale terpenoid production by marine microalgae suggests that thraustochytrids could serve also for large-scale commercial generation of squalene [32].

The present study evaluated the potential production of DHA and squalene by the marine thraustochytrid species *Aurantiochytrium* sp. T66 (ATCC PRA-276). Heterotrophic cultivation of *Aurantiochytrium* sp. was initially carried out on pure glucose and was then replaced by forest biomass hydrolysates (enzymatically saccharified organosolv-pretreated birch chips). To the best of our knowledge, this is the first report describing the use of forest biomass for the cultivation of a thraustochytrid species and the consequent co-production of DHA and squalene. The proposed strategy is promising for the commercial production of various value-added compounds.

## Results and discussion

### Batch cultivations of *Aurantiochytrium* sp. T66 in flask and bioreactor

*Aurantiochytrium* sp. is a heterotrophic unicellular marine thraustochytrid, closely related to heterokont algae and well known for its elevated DHA production [33]. It is ubiquitous in marine environments such as mangroves and mud flats, feeding mainly on organic substrates present in the environment [17]. It can grow on various types of sugars, but glucose is known to support high cell density along with high amounts of DHA [34]. It can tolerate high concentrations of carbon sources, including as much as 120 g/L of glucose [35]. In the present study, we evaluated the DHA and squalene biosynthetic ability of this strain during batch cultivation on glucose (30 g/L) derived from organosolv-pretreated birch hydrolysate (OPBH), both in flasks and bioreactors.

**Fig. 1:**
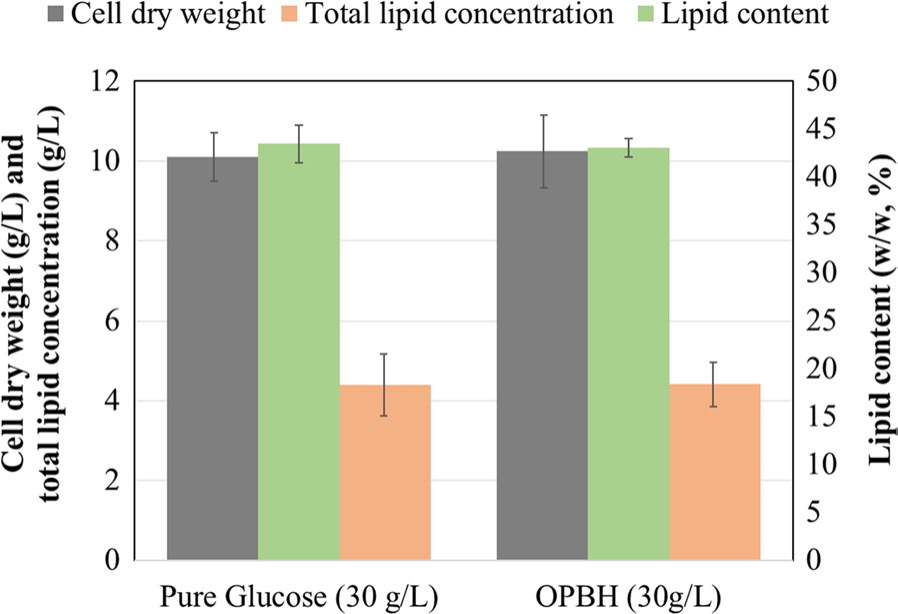
Comparison of *Aurantiochytrium* sp. cultivated on pure glucose and OPBH. Growth and lipid production by *Aurantiochytrium* sp. T66 (ATCC PRA-276) grown in Erlenmeyer flasks on either pure glucose or OPBH containing 30 g/L of glucose

Growth on commercial glucose (30 g/L) in a flask was assessed first and compared with results on OPBH. Cell dry weight (CDW), lipid concentration, DHA content, and squalene concentration were similar between an OPBH-grown culture and those cultivated on pure glucose (Fig. 1), as can be expected given the absence of inhibitors in the hydrolysate [36]. Hybrid organosolv-steam explosion was used to treat the birch biomass, resulting in high-cellulose-containing solids (89%, w/w), which were enzymatically saccharified at 10% w/w solids content, yielding 77.07 g/L of glucose [37]. Yeast extract was used as nitrogen source (11.6% w/w total nitrogen). Yeast extract (containing 9–12% of total nitrogen) is usually obtained from spent yeast biomass of brewery industries and is commonly used for large-scale growth of thraustochytrid species including *Aurantiochytrium* sp. [38].

**Fig. 2:**
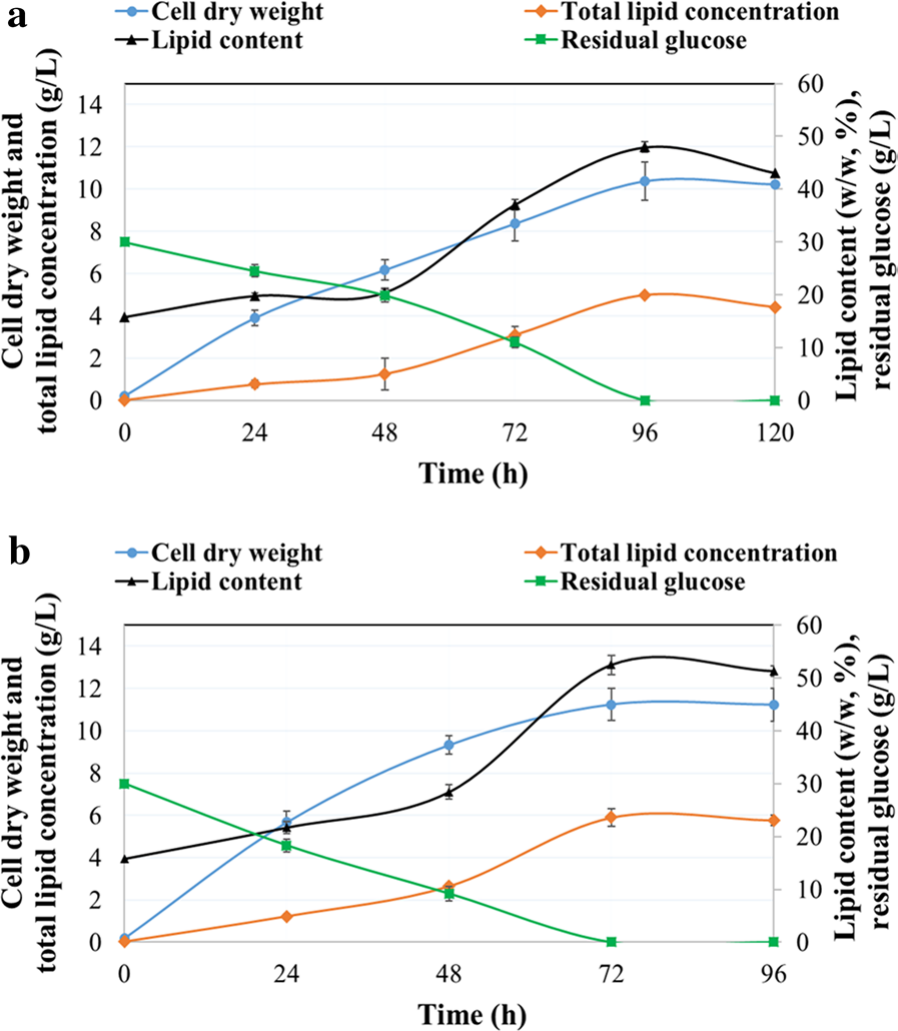
Batch cultivation of *Aurantiochytrium* sp. in flask and bioreactor. Time-course determination of cell dry weight (g/L), total lipid concentration (g/L), lipid content (%, w/w), and residual glucose (g/L) of *Aurantiochytrium* sp. T66 (ATCC PRA-276) cultivated on OPBH containing 30 g/L of glucose in **a** erlenmeyer flasks and **b** bioreactor

**Fig. 3:**
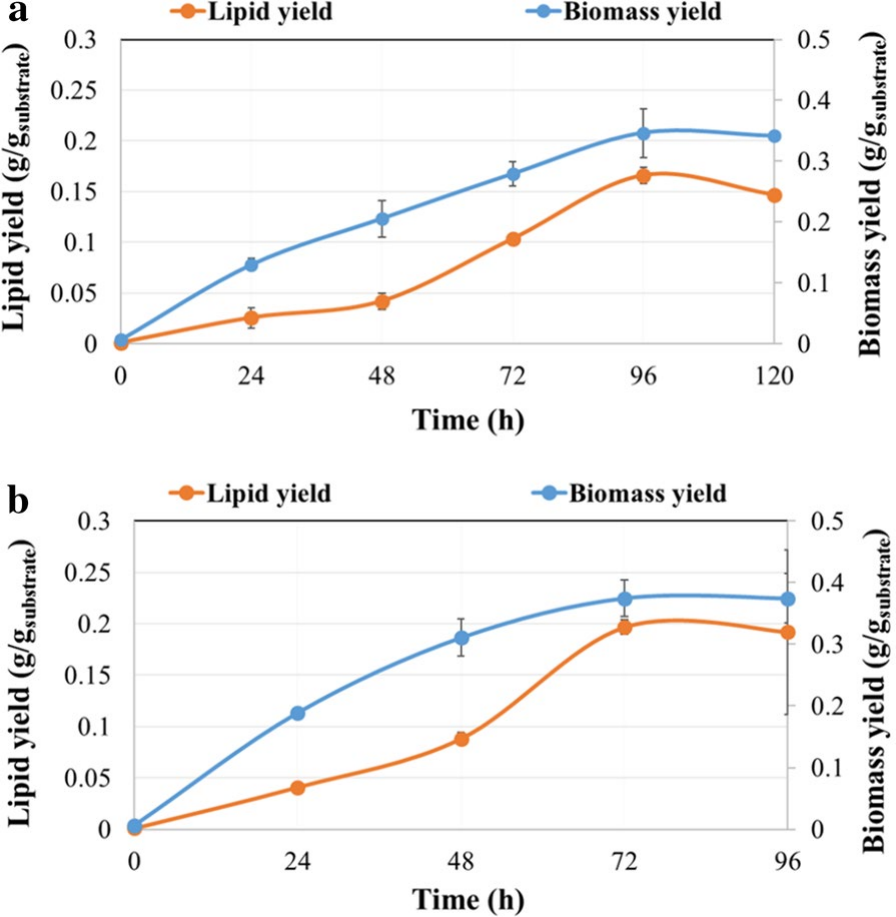
Estimation of biomass and lipid yield of *Aurantiochytrium* sp. cultivated in flask and bioreactor. Time-course determination of biomass yield (g/g_substrate_) and lipid yield (g/g_substrate_) of *Aurantiochytrium* sp. T66 (ATCC PRA-276) cultivated on OPBH containing 30 g/L of glucose in **a** erlenmeyer flasks and **b** bioreactor

Time-course results showing cell dry weight (g/L), total lipid concentration (g/L), lipid content (% w/w), and residual glucose (g/L) following growth of the microalga in flask or bioreactor are presented in Fig. 2. Both flask and bioreactor cultures were inoculated with a 2-day grown seed culture and 15 mL were harvested immediately after inoculation; this resulted in 0.19 ± 0.02 g/L cell dry weight at 0 h of cultivation. After 24 h, cell dry weight was 3.89 ± 0.13 g/L in flask and 5.67 ± 0.55 g/L in bioreactor cultures, with glucose consumption being 5.46 g/L and 11.68 g/L, respectively. The corresponding total lipid concentration was 0.77 ± 0.17 g/L and 1.23 ± 0.13 g/L (Fig. 2). The exponential phase of algal growth was reached within the first 8 h of cultivation, as observed by OD_600_ values (data not reported here). After 24 h, growth continued linearly until the stationary phase was reached at 96 h in flasks and 72 h in the bioreactor (Fig. 2). A similar growth profile had been observed with other species, such as *Aurantiochytrium* SW1 [39] and *Aurantiochytrium* sp. SD116 [40]. The highest cell dry weight for *Aurantiochytrium* sp. cultivated in flask (10.39 ± 0.73 g/L) was attained after 96 h, with the supplied glucose being almost completely consumed by that point (Fig. 2a). At the same time, maximum total lipid concentration was 4.98 ± 0.38 g/L, or 47.93% (w/w) of lipid content (Fig. 2a). In contrast, in the bioreactor, the highest cell dry weight (11.24 ± 0.79 g/L) was attained after 72 h of cultivation and no glucose was left in the medium at that point (Fig. 2b). The maximum lipid concentration in the bioreactor was 5.9 ± 0.43 g/L, or 52.49% (w/w) of lipid content (Fig. 2b). The observed lipid synthesis pattern was similar to that of other heterotrophically cultivated microalgae such as *Crypthecodinium cohnii*, and other thraustochytrids such as *Schizochytrium* sp. S31 [41, 42]. Both of these strains are also well studied for their DHA production [43]. In another study, strain *Aurantiochytrium* sp. ATCC PRA-276 generated 9.3 g/L of cell dry weight when cultivated on 30 g/L of glucose with 3 g/L of total nitrogen (1.36 g/L (NH_4_)_2_SO_4_, 13.63 g/L yeast extract, and 13.63 g/L monosodium glutamate), whereas a higher cell dry weight was observed when the strain was cultivated on 0.44 g/L of total nitrogen (0.2 g/L (NH_4_)_2_SO_4_, 2.0 g/L yeast extract, and 2.0 g/L monosodium glutamate) with the same amount of glucose [44]. Other thraustochytrid species such as *Schizochytrium* sp. S31 exhibited a similar pattern of glucose utilization, by utilizing all the glucose present in medium after 5 days [45]. *Schizochytrium* sp. S31 synthesized 6.01 g/L of biomass and 2.38 g/L of total lipids, corresponding to 39.60% w/w lipid content when cultivated in a flask at pH 7.0 with 20 g/L glucose and 4 g/L of yeast extract [45]. After reaching stationary phase, cell dry weight of *Aurantiochytrium* sp. declined slightly, mainly due to fewer lipids (Fig. 2). At this point, the cells were harvested; alternatively, the lipids could be utilized for further growth. Once they have exhausted all carbon sources from the medium, oleaginous microorganisms can utilize lipid reserves for growth and biomass production through β-oxidations of fatty acids [46]. Lipid degradation was observed also in *Aurantiochytrium* sp. 18 W-13a, in which lipids dropped drastically from 3.90 ± 0.22 to 1.53 ± 0.21 g/L between the 4th and 8th day of cultivation after glucose exhaustion [47]. The results of biomass and lipid formation yield are presented in Fig. 3. Maximum biomass and lipid formation yield of 0.35 g/g_substrate_ and 0.17 g/g_substrate_, respectively, were obtained after 96 h of cultivation in flask (Fig. 3a). In bioreactors, higher biomass and lipid yield of 0.37 g/g_substrate_ and 0.19 g/g_substrate_, respectively, were obtained at 72 h (Fig. 3b). Biomass productivity was 2.59 g/L/day and 3.74 g/L/h for cultures grown in flasks and bioreactor, respectively; while the corresponding lipid productivity was 1.25 g/L/day and 1.97 g/L/day (Table 1). The higher yield and productivity obtained in the bioreactor were likely a result of better pH regulation and oxygen transfer during growth [17].

**Table 1:** Summary of various parameters relating to the cultivation of *Aurantiochytrium* sp. T66 on OPBH in flask and bioreactor

Parameters	Cultivation in flask	Cultivation in bioreactor
Cell dry weight (g/L)	10.39 ± 0.73	11.24 ± 0.79
Biomass yield (g/g_substrate_)	0.35 ± 0.02	0.37 ± 0.01
Biomass productivity (g/L/day)	2.59 ± 0.12	3.74 ± 0.14
Total lipid concentration (g/L)	4.98 ± 0.38	5.90 ± 0.43
Lipid content (%, w/w)	47.93 ± 0.96	52.49 ± 0.92
Lipid yield (g/g_substrate_)	0.17 ± 0.01	0.20 ± 0.02
Lipid productivity (g/L/day)	1.25 ± 0.06	1.97 ± 0.05
DHA content (%, w/w_total lipids_)	25.98 ± 1.20	35.76 ± 0.98
DHA concentration (g/L)	1.29 ± 0.16	2.11 ± 0.09
DHA yield (mg/g_CDW_)	124.15 ± 5.45	187.22 ± 3.21
DHA productivity (mg/L/day)	322.50 ± 12.34	703.33 ± 10.23
Squalene yield (mg/g_CDW_)	69.31 ± 6.43	88.47 ± 7.51
Squalene concentration (g/L)	0.72 ± 0.02	1.0 ± 0.03

### Effect of cultivation conditions on DHA concentration of *Aurantiochytrium* sp.

**Fig. 4:**
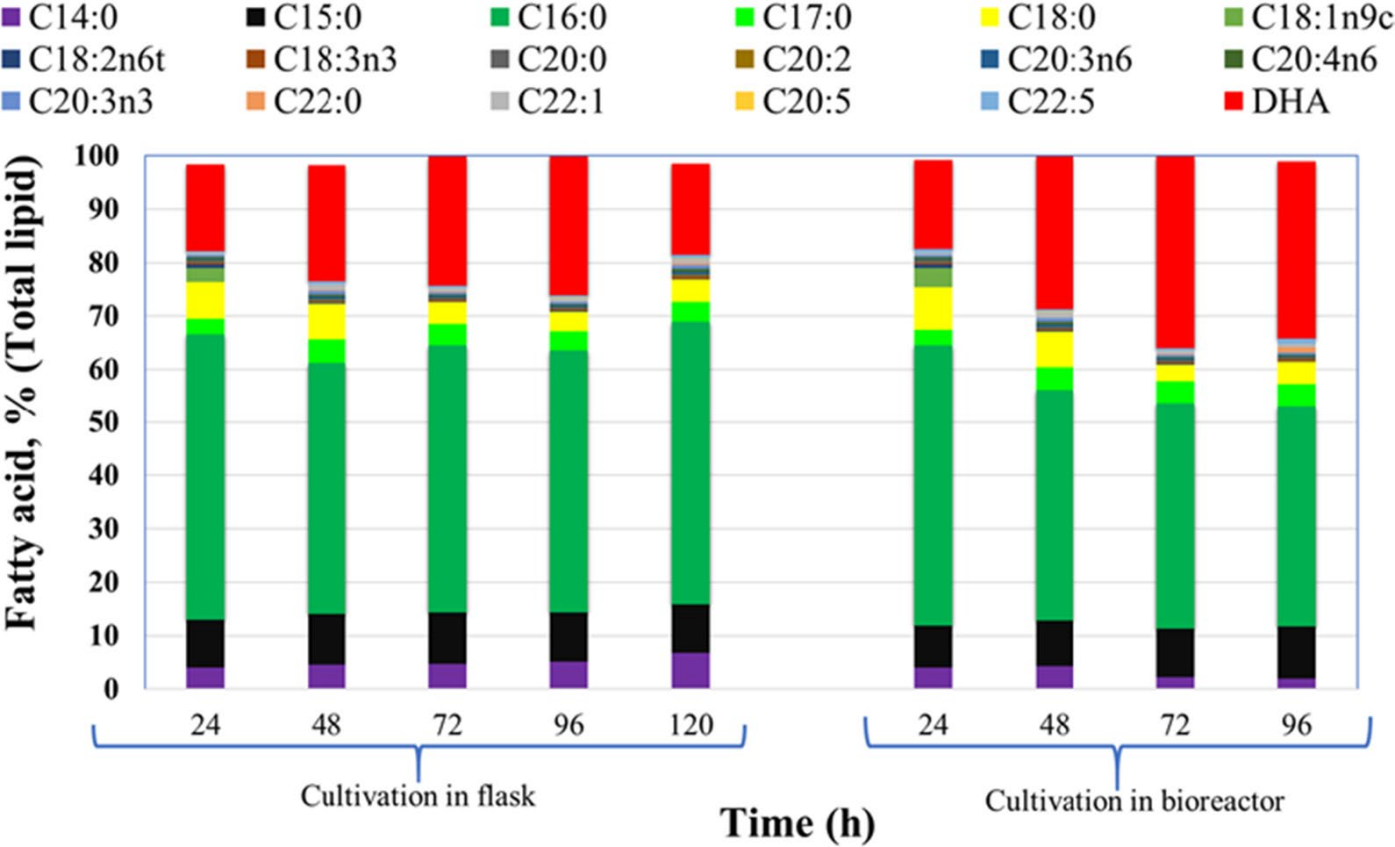
Estimation of the fatty acid profile of *Aurantiochytrium* sp. Fatty acid profile (% w/w_total lipid_) of *Aurantiochytrium* sp. T66 (ATCC PRA-276) cultivated on OPBH containing 30 g/L of glucose in erlenmeyer flasks and bioreactor

**Fig. 5:**
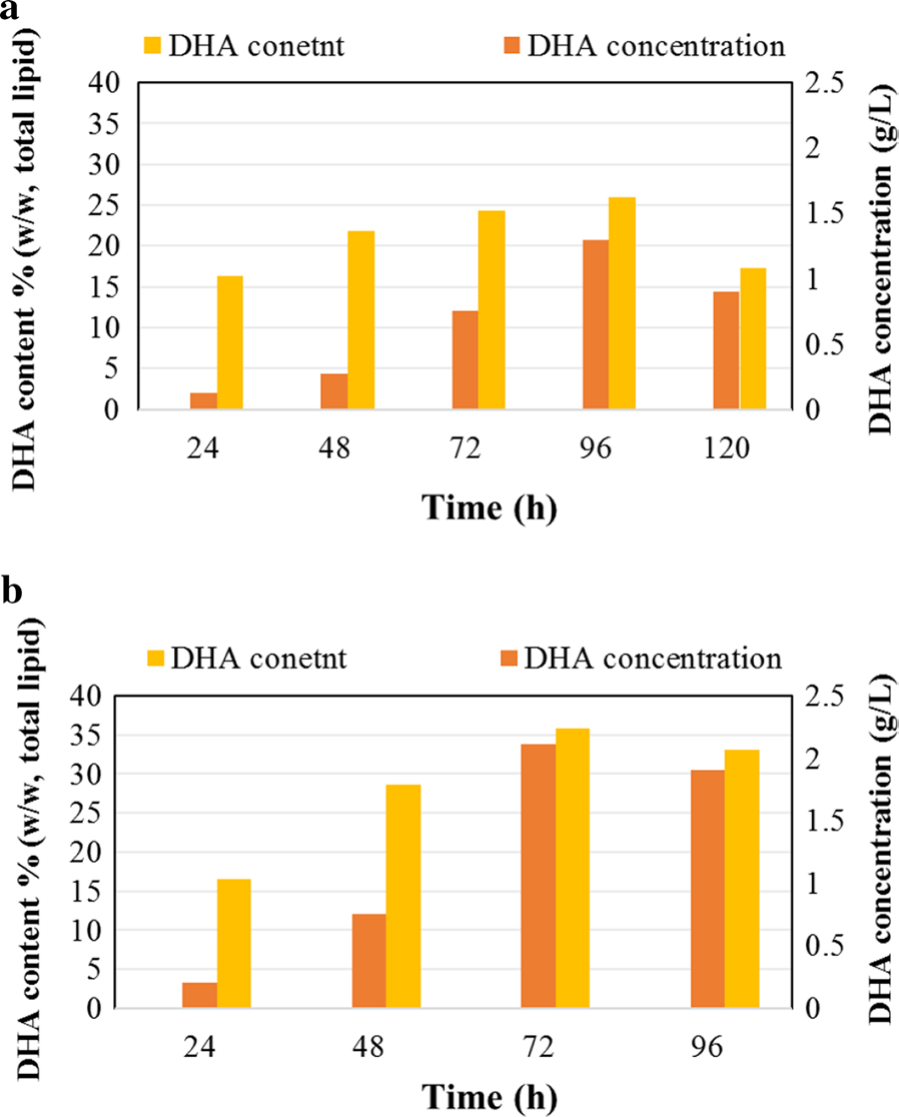
Quantification of DHA in extracted lipids. Estimation of DHA concentration (g/L) and DHA content (%, w/w_total llipids_) of *Aurantiochytrium* sp. T66 (ATCC PRA-276) cultivated on OPBH containing 30 g/L of glucose in **a** erlenmeyer flasks and **b** bioreactor

The fatty acid profile of *Aurantiochytrium* sp. is presented in Fig. 4 and is similar to that of other thraustochytrids [48]: the main fatty acids were C_14:0_, C_15:0_, C_16:0_, C_17:0_, C_18:0_, and DHA, and their ratio changed with cultivation time. When this strain was cultivated to stationary phase (96 h) in a flask, the following amounts of lipids were synthesized: C_14:0_ (5.17%), C_15:0_ (9.21%), C_16:0_ (49.19%), C_17:0_ (3.60%), C_18:0_ (3.59%), and DHA (25.98%). When cultivated for 72 h in a bioreactor, the following amounts were synthesized: C_14:0_ (2.17%), C_15:0_ (9.00%), C_16:0_ (42.32%), C_17:0_ (4.08%), C_18:0_ (3.09%), and DHA (35.76%). Time-course experiment data regarding DHA concentration (g/L) and DHA content (%, w/w of total lipids) of *Aurantiochytrium* sp. cultivated in a flask and bioreactor are presented in Fig. 5a. *Aurantiochytrium* sp. synthesized 16.28% DHA (w/w_total lipids_) after 24 h of cultivation in a flask (Fig. 4a); the same amount was obtained after only 24 h in a bioreactor (16.56% w/w_total lipids_) and using OPBH as a substrate (Fig. 5b). When the algae were cultivated in flasks, the highest DHA content was 25.98% w/w_total lipids_ after 96 h, corresponding to a DHA concentration of 1.29 g/L. Further extension of the growth period to 120 h resulted in reduced DHA content and concentration: 17.23% w/w_total lipids_ and 0.89 g/L, respectively. When the algae were cultivated in a bioreactor, considerably higher DHA content and concentration were achieved at an earlier time (72 h): 35.76% w/w_total lipids_ and 2.1 g/L, respectively (Fig. 5b). The high quantity of DHA in the bioreactor-grown culture resulted most likely from improved oxygen supply during growth as the conversion of saturated fatty acids into unsaturated fatty acids proceeds at higher rates when oleaginous microorganisms are grown under abundant oxygen [17]. DHA production by several thraustochytrids on different media is presented in Table 2. Optimized conditions lead to a high PUFA yield [49]. *Thraustochytrium* sp. ATCC 26185 previously showed enhanced DHA production, from 1.16 to 1.68 g/L, after optimization of various fermentation parameters, such as initial pH (6.89), inoculum volume (4.16%), and fermentation volume (140.47 mL) [50]. Moreover, cultivation of thraustochytrid strains in flasks resulted in significantly different levels of DHA production compared to bioreactor cultivation. For example, *Thraustochytrium roseum* exhibited enhanced growth in flasks compared to a stirred tank bioreactor, in which growth was inhibited due to high mechanical stirring [51]. In contrast, *Schizochytrium* sp. SR21 exhibited better growth in a bioreactor than in flasks because this strain was highly resistant to mechanical stirring [52]. Three species of *Schizochytrium mangrovei* (FB1, FB2, and FB3) cultivated on 20 g/L of glucose in a flask synthesized from 32.29 to 39.14% w/w_total lipids_ of DHA, and only a slight difference in fatty acids composition (C14:0, C16:0, C22:5, and C22:6) was observed when the culture was harvested in either early or late stationary phase [53].

**Table 2:** Production of DHA by various marine thraustochytrids

Microorganisms	Cultivation mode	Glucose	Nitrogen	Cell dry weight (g/L)	Lipid concentration (g/L)	DHA concentration (%, total lipid)	References
*Aurantiochytrium* sp. ATCC PRA-276	Batch in flask	Glucose (30 g/L)	3.0 g/L (1.36 g/L (NH_4_)_2_SO_4_, 13.63 g/L yeast extract and 13.63 g/L monosodium glutamate)	9.3	–	5.5	[44]
			0.44 g/L (0.1 g/L de (NH4)2SO4, 1.0 g/L yeast extract, 1.0 g/L monosodium glutamate)	17.0	–	12.5	
*Aurantiochytrium* sp. KRS101	Baffled flask	Orange peel extract glucose (5.9 g/L), fructose (5.6 g/L), organic acids	NaNO_3_ (1.2 g/L)	4.40	2.12 (FAME)	14.31	[62]
		5 g/L glucose + orange peel extract glucose (5.9 g/L), fructose (5.6 g/L), organic acids	NaNO_3_ (1.5 g/L)	5.50	2.85	14.18	
*Aurantiochytrium* sp. KRS101	Flask	Modified basal medium glucose (60 g/L)	Corn steep solid	9.00	3.42	19.88	[34]
*Schizochytrium limacinum* SR 21	Flask	Glucose (90 g/L)	Yeast extract (2 g/L)	18.2	24.2	14.72	[63]
		Glycerol (100 g/L)	Yeast extract (2 g/L)	20.10	15.03	18.38	
*Aurantiochytrium* 4 W-1b	Flask	Glucose (30 g/L)	1% tryptone and 0.5% yeast extract	9.01 ± 0.62	4.16 ± 0.48	27.9	[35]
*Aurantiochytrium* SW1	Flask	Fructose (70 g/L)	10 g/L monosodium glutamate	19.0	9.13	25	[39]
*Aurantiochytrium* sp. T66 (ATCC PRA-276)	Flask	Organosolv-pretreated birch hydrolysate (30 g/L glucose)	Yeast extract (C:N, 10)	10.39 ± 0.73	4.98 ± 0.38	25.98 ± 1.20	This study
	Bioreactor			11.24 ± 0.79	5.90 ± 0.43	35.76 ± 0.98	

### Extraction of squalene from *Aurantiochytrium* sp. cultivated in flask and bioreactor

The light orange color of the cultures indicated the presence of carotenoids, which have antioxidant activity, in the cells of *Aurantiochytrium* [54]. Squalene, astaxanthin, echinenone and lutein are the most important carotenoids present in *Aurantiochytrium* [17]. Squalene production by other *Aurantiochytrium* strains is presented in Table 3.

**Table 3:** Squalene production by various marine thraustochytrids

Microorganisms	Feedstock	Squalene concentration (mg/g_CDW_)	References
*Schizochytrium mangrovei* FB1	Glucose (20 g/L)	0.162	[53]
*S. mangrovei* FB2		~ 0.08	
*S. mangrovei* FB3		~ 0.05	
*S mangrovei*	Glucose (20 g/L) + methyl jasmonate (0.1 mM)	1.17	[56]
*Aurantiochytrium* sp. BR-MP4-A1	Glucose (20 g/L)	0.72	[64]
*Aurantiochytrium* sp. 18 W-13a	Glucose (20 g/L)	198	[47]
*Aurantiochytrium* sp. 18 W-13a	Glucose (20 g/L)	171.1 ± 6.7	[57]
*Aurantiochytrium* sp. T66 (ATCC PRA-276)	Organosolv-pretreated birch hydrolysate (30 g/L glucose)	69.31 ± 6.43 (flask cultivation)	This study
		88.47 ± 7.51 (bioreactor cultivation)	

**Fig. 6:**
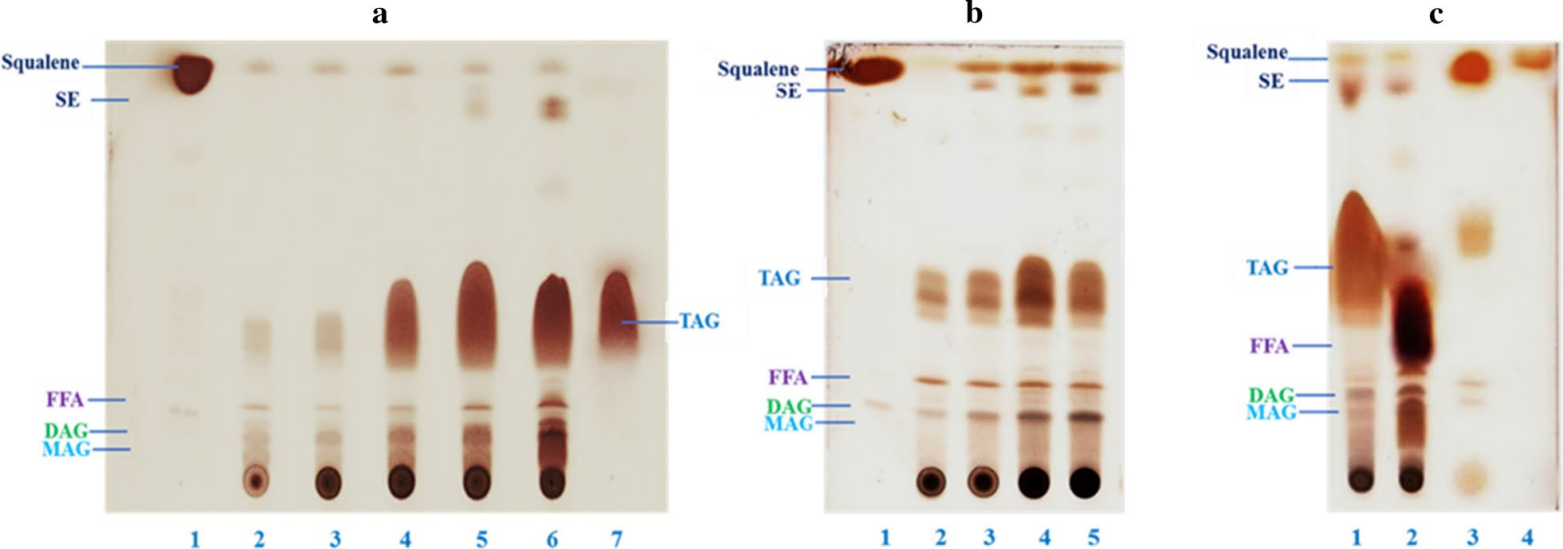
TLC analysis of extracted lipids from *Aurantiochytrium* sp. Analysis of squalene in total lipid extracts from *Aurantiochytrium* sp. T66 (ATCC PRA-276) cultivated on OPBH containing 30 g/L of glucose in **a** erlenmeyer flasks and **b** bioreactor. **a** Lane 1, squalene standard; lanes 2–6, samples cultivated for 24–120 h; lane 7, TAG standard. **b** Lane 1, squalene standard; lanes 2–5, samples cultivated for 24–96 h. **c** Purification of squalene from total lipid extracts following saponification: lane 1, unsaponifiable fraction; lane 2, saponified fraction; lane 3, purified product from unsaponifiable fraction; lane 4, squalene standard

**Fig. 7:**
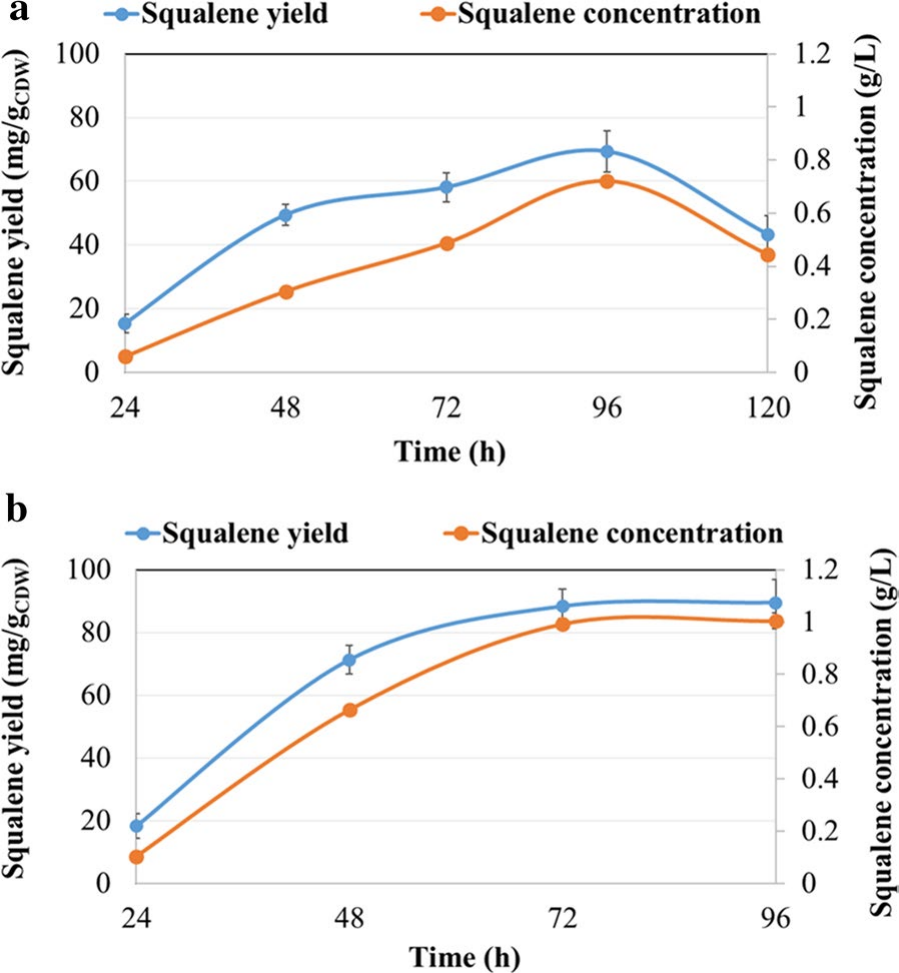
Estimation of squalene concentration in extracted lipids from *Aurantiochytrium* sp. Evolution of squalene yield (mg/g_CDW_) and concentration (g/L) during cultivation of *Aurantiochytrium* sp. T66 (ATCC PRA-276) on OPBH containing 30 g/L of glucose in **a** erlenmeyer flasks and **b** bioreactor

Squalene is an intermediate of the cholesterol biosynthesis pathway and acts as a natural antioxidant to protect the cells form reactive oxygen species [24]. The qualitative evaluation of squalene in total lipids was carried out by thin-layer chromatography (TLC) and the chromatograms corresponding to flask and bioreactor-grown cultures are presented in Fig. 6a, b, respectively. Squalene concentration (g/L) and yield (mg/g_CDW_) of *Aurantiochytrium* sp. cultivated on OPBH in a flask or bioreactor are presented in Fig. 7a, b, respectively. Squalene concentration increased from 0.05 g/L (15.34 mg/g_CDW_) to 0.72 g/L (69.31 mg/g_CDW_) when algae were cultivated in flasks for 96 h instead of 24 h. Extending growth to 120 h had a negative impact on squalene concentration, which dropped to 0.44 g/L (43.35 mg/g_CDW_). This is likely due to increased synthesis of steryl ester (SE) from squalene once stationary phase was reached, as shown in the Fig. 6a, lane 6. During bioreactor cultivation, the concentration of squalene increased from 0.10 g/L (16.56 mg/g_CDW_) to 1.0 g/L (88.47 mg/g_CDW_) between 24 and 96 h. Squalene was clearly visible in the total lipid extract obtained from algae cultivated in a bioreactor for 24 h to 96 h (Fig. 6b, lanes 2 to 5). Squalene purification was carried out by fractionating total lipids into saponifiable and unsaponifiable fractions and was followed by TLC analysis. As shown in Fig. 6c, the unsaponifiable fraction presented a high amount of squalene (Fig. 6c, lane 1); whereas the saponifiable fraction presented only a minor yet easily detected amount (Fig. 6c, lane 2). Following saponification of total lipids, squalene was extracted from the total unsaponifiable lipids fraction with *n*-hexane as solvent. After solvent evaporation, the colorless liquid was identified by TLC (Fig. 6c, lane 3) using a squalene standard (Fig. 6c, lane 4). The squalene extracted from the unsaponifiable fraction was not 100% pure, as it contained a minor amount of triacylglycerol (TAG, Fig. 6c, line 3). Further purification can be achieved by fractional crystallization or other column chromatographic methods [31, 53].

Nakazawa et al. [55] screened 176 strains isolated from Japan and other Asian countries to determine squalene accumulation using TLC. Among the 176 strains, only 38 were capable of synthesizing high amounts of squalene [55]. In another study, Jiang et al. [53] cultivated three species of *S. mangrovei* (FB1, FB2, and FB3) on 20 g/L of glucose in flask to study fatty acid composition and squalene content. The highest squalene content of 0.162 mg/g_CDW_ (8.53 g/L CDW) was reported in *S. mangrovei* FB1. In another study, the same group studied the effect of adding methyl jasmonate (MJA) in the range 0–0.4 mM: addition of 0.1 mM MJA resulted in 1.17 ± 0.006 mg/g_CDW_ of squalene at 48 h of cultivation, or 60% more than in the control [56]. The authors suggested that this result depended on increased squalene synthase activity. However, squalene content in these strains was extremely low compared to that of other thraustochytrids such as *Aurantiochytrium* sp. 18W-13a, which accumulated 198 mg/g_CDW_ (1.29 ± 0.13 g/L) of squalene at 4 days of cultivation on 20 g/L of glucose, 1% proteose-peptone, and 0.5% yeast extract [47]. In another study, squalene content from the same strain was optimized by varying temperature, salinity, and glucose concentration. Specifically, the highest amount of squalene was 171 mg/g_CDW_ (0.9 g/L) when *Aurantiochytrium* sp. 18W-13a was cultivated on glucose-peptone-yeast medium containing 20 g/L of glucose with 25 to 50% of seawater at 25 °C [57]. Besides naturally occurring strains, genetic engineering has allowed the construction of strains capable of producing squalene. For example, the oleaginous yeast *Yarrowia lipolytica* was genetically engineered by deleting the ggs1 gene which was integrated at the YALI0F30987g locus in the CIBTS2112 strain. The ensuing CIBTS2112ΔG strain synthesized 44.5 ± 1.9 mg/g_DCW_ (531.6 ± 25 mg/L) of squalene, a 4.5-fold increase over the parental strain CIBTS2112 [58] or 1.75-fold that of other engineered oleaginous yeast strains [59, 60].

Furthermore, a comprehensive overview of various parameters, including cell dry weight, lipid concentration, lipid content, biomass and lipid yield, their productivity, DHA content and concentration, squalene yield and concentration of *Aurantiochytrium* sp. T66 cultivated on OPBH in flask or bioreactor is provided in Table 1.

## Conclusion

The present study shows that *Aurantiochytrium* sp. T66 is a promising candidate for commercial DHA production. Importantly, this strain has strong potential to co-synthesize DHA and squalene, which could be more economical for the pharmaceutical and nutraceutical industries. Specifically, the heterotrophic cultivation of *Aurantiochytrium* sp. T66 on organosolv-pretreated birch hydrolysate instead of pure glucose suggests more economically viable production of nutraceuticals due to consumption of sustainable and cost-effective non-edible lignocellulosic feedstocks. To this end, the highest DHA concentration (2.11 g/L) and DHA content (35.76% w/w_total lipids_) were observed after 96 h of cultivation in a bioreactor. Under these conditions, DHA productivity was 703.33 ± 10.33 mg/L/day and squalene reached a total of 1.0 ± 0.03 g/L (88.47 ± 7.51 mg/g_CDW_).

## Methods

### Microalgal strain and cultivation conditions

*Aurantiochytrium* sp. T66 (ATCC PRA-276) was procured from the American Type Culture Collection (ATCC). *Aurantiochytrium* sp. T66 was maintained on ATCC 2673 Thraustochytrid medium containing yeast extract (1 g/L), peptone (15 g/L), and glucose (20 g/L) in artificial sea water. The pH of the medium was adjusted to 6.8 before autoclaving. The artificial seawater consisted of (g/L): NaCl, 18; MgSO_4_·7H_2_O, 2.44; KCl, 0.6; NaNO_3_, 1.0; CaCl_2_·2H_2_O, 0.3; KH_2_PO_4_, 1.0; Tris buffer, 1.0; NH_4_Cl, 0.027; Vitamin B_12_, 15 × 10^−8^; chelated iron solution, 3 mL; and metal solution, 10 mL. The chelated iron solution and metal solution were prepared as described by UTEX Culture Collection of Algae at the University of Texas at Austin [61]. Seed cultures were grown in the aforementioned medium in 250-mL Erlenmeyer flasks with 100 mL of medium and were incubated at 25 °C with continuous shaking at 180 rpm.

### Batch cultivation of *Aurantiochytrium* sp. using organosolv-pretreated birch hydrolysate in flask and bioreactor

Silver birch (*Betula pendula* L.) was pretreated according to our previously mentioned protocol [36]. Briefly, silver birch wood chips milled to < 1 mm were pretreated in a hybrid organosolv-steam explosion reactor at 200 °C with 60% v/v ethanol and 1% w/w_biomass_ H_2_SO_4_ for 15 min. The pretreated solids were removed from the pretreatment liquor by vacuum filtration, air-dried, and enzymatically saccharified at 10% w/w solids with 20 FPU/g_solids_ of Cellic CTec2 (Novozymes A/S, Bagsværd, Denmark) in 50 mM citrate–phosphate buffer (pH 5) for 48 h at 50 °C with continuous shaking [36, 37]. At the end of enzymatic saccharification, the remaining solids were removed by centrifugation and the hydrolysate was used for subsequent cultivation trials.

Growth medium was prepared in 50% artificial seawater, by adding the appropriate amount of OPBH to yield 30 g/L of glucose and an appropriate amount of yeast extract to maintain a C:N ratio of 10:1 (g/g) (with yeast extract having 11.6% of total nitrogen, based on the certificate of analysis provided by Sigma-Aldrich, St. Luis, MO, USA). To avoid sugar degradation, an appropriate amount of OPBH in distilled water and seawater containing yeast extract were autoclaved separately and mixed after being cooled to room temperature. The pH of the medium was maintained at 6.8 before autoclaving. Initially, the cultivations were carried out in 250-mL Erlenmeyer flasks containing 100 mL of medium, which was inoculated with 10% v/v of seed culture and incubated at 25 °C and 180 rpm. Time-course experiments were performed by sampling 10 mL of culture every 24 h and were used to determine cell dry weight (g/L), lipid concentration (g/L), lipid content (%, w/w), residual glucose (g/L), DHA concentration (%), and squalene content (mg/g_CDW_). Thereafter, *Aurantiochytrium* sp. was cultivated in a 1-L bioreactor (Bio console ADI1025, Applikon Biotechnology, JG Delft, The Netherlands) with 300 mL of OPBH medium (30 g/L of glucose) in 50% artificial seawater and yeast extract (C:N_g/g_, 10). The pH was controlled at 6.8 with 3 N NaOH and 3 N HCl and temperature was maintained at 25 °C. During the experiment, dissolved oxygen was maintained above 50% of saturation by sparging with compressed air. Aliquots of 15 mL were sampled every 24 h to estimate residual glucose concentration, cell dry weight, lipid concentration, and squalene concentration.

### Estimation of cell dry weight, lipid concentration, and residual glucose in a time-course experiment

To separate the cells from the medium, the samples were centrifuged at 8000 rpm for 10 min. The supernatant was kept for residual sugars determination, whereas the pellet was washed with distilled water to remove media components. The cell pellet was dried in pre-weighed aluminum boats in a hot-air oven at 40 °C until constant weight was attained. Cell dry weight (g/L) was determined gravimetrically. Subsequently, dried biomass was crushed into paste/fine powder by mortar and pastel and mixed with chloroform and methanol (2:1, v/v). The slurry was incubated for 2 h with continuous shaking at room temperature and finally ½ the volume of water was added to the slurry for phase separation. The bottom chloroform layer was aspirated with a pipette in pre-weighed glass vials. Total lipids (g/L) were determined gravimetrically and lipid content (% w/w) was estimated by the following formula;

$${\text{Lipid}}\,{\text{content}} \left( {\% ,\frac{w}{w}} \right) = \left( {{\raise0.7ex\hbox{${{\text{Total}}\,{\text{lipid}}\,{\text{concentration}} }$} \!\mathord{\left/ {\vphantom {{{\text{Total}}\,{\text{lipid}}\,{\text{concentration}} } {{\text{Cell}}\,{\text{dry}}\,{\text{weight}}}}}\right.\kern-0pt} \!\lower0.7ex\hbox{${{\text{Cell}}\,{\text{dry}}\,{\text{weight}}}$}}} \right) \times 100$$

Residual glucose was analyzed on a high-performance liquid chromatographer equipped with an Aminex HPX-87H column (Bio-Rad, Hercules, CA, USA) and a refractive index detector. The column was maintained at 65 °C and 0.6 mL/min of 5 mM H_2_SO_4_ was used as the mobile phase.

### Extraction and purification of squalene from total lipids

Squalene was extracted from total lipids following the protocol suggested by Nakazawa et al. [55]. Total extracted lipids were hydrolyzed using 0.5 M ethanolic (75%, v/v) KOH solution in a three-necked flask equipped with a reflex condenser at 90 °C for 1 h. The mixture was cooled down at room temperature and non-saponifiable lipids were separated from saponifiable lipids by three rounds of extraction with *n*-hexane. Total *n*-hexane was aspirated in a pre-weighed glass vial and evaporated until constant weight was attained. Squalene content (mg/g_CDW_) was determined gravimetrically.

### Determination of squalene by thin-layer chromatography

Total lipids were analyzed for squalene content by TLC on silica gel 60 plates using a mobile phase consisting of *n*-hexane:diethyl ether:acetic acid (85:15:1; v/v/v). The separated bands were visualized by spraying methanolic MnCl_2_ solution containing MnCl_2_·4H_2_O (0.32 g), water (30 mL), methanol (30 mL), and concentrated H_2_SO_4_ (4 mL). The plate was charred in a hot-air oven at 125 °C for 5 min. The squalene band in total lipids was estimated using pure squalene (≥ 98%, liquid, S3626, Sigma-Aldrich) as standard.

### Quantification of squalene by high-performance liquid chromatography (HPLC)

A standard stock solution of squalene at 10 mg/mL was prepared with pure squalene in absolute ethanol (≥ 99.8%). The standard calibration curve was prepared using squalene concentrations ranging from 0.001 to 10 mg/mL in acetonitrile. The analysis was performed by HPLC (PerkinElmer, Waltham, MA, USA) using a C18 reverse-phase end-capped chromatography column (NUCLEOSIL^®^ 100-5 C18, 5 µm particles, 100 Å pores, 50 mm, MACHEREY–NAGEL GmbH & Co. KG, Düren, Germany) operating at 30 °C with HPLC-grade acetonitrile:water (9:1, v/v) as mobile phase at a flow rate of 1.5 mL/min with 20 μL of injection volume. The squalene was detected with a UV detector at 210 nm. Samples of lipid-containing squalene were dissolved in 100% acetonitrile.

### Estimation of fatty acid profiles and DHA in total extracted lipids

Estimation of the fatty acid profile was carried out after transesterification of extracted lipids using acid catalysts (8 mL of 6% methanolic H_2_SO_4_). Fatty acid methyl esters (FAMEs) were extracted with *n*-hexane and analysis was performed with a GC-FID chromatographer (Agilent, Santa Clara, CA, USA) equipped with a capillary column (Select FAME; dimensions 50 m × 0.25 mm ID and 0.25 μm film thickness). Supelco 37 Component FAME Mix (47885-U, Sigma-Aldrich) was used as a standard to identify the peaks of FAMEs.

### Statistical analysis

The experimental values are presented as mean ± standard deviation, with all the trials to be performed in three replicates. Microsoft Office Excel 2016 (Microsoft, USA) was used for one-way analysis of variance (ANOVA) with *p* < 0.05 for data acceptance.

## Data Availability

The materials produced during the current study are available from the corresponding author on reasonable request.
